# Comparison of Chlorhexidine, Chlorhexidine with anti-discoloration system, and Polyvinylpyrrolidone-iodine on early wound healing after dental implant placement: A randomized clinical trial

**DOI:** 10.1007/s00784-025-06665-y

**Published:** 2025-11-28

**Authors:** Malin Strasding, Marcus Eberhardt, Stefan Paul Hicklin, Patrick R. Schmidlin, Philipp Sahrmann

**Affiliations:** 1 Center of Dental Medicine, Division of Periodontology and Peri-Implant Diseases, Clinic of Conservative and Preventive Dentistry, University of Zurich, Zurich, Switzerland; 2 Division of Fixed Prosthodontics and Biomaterials, University Clinic of Dental Medicine, University of Geneva, Rue Michel-Servet 1, CH-1211 Geneva, Switzerland; 3 Zahnarztzentrum.Ch, Baden, Switzerland; 4 Department of Periodontology, Endodontology and Cariology, University Center for Dental Medicine Basel UZB, University of Basel, Basel, Switzerland

**Keywords:** Antiseptic mouth rinse, Chlorhexidine, Anti-discoloration system, Povidone-iodine, Dental implants, Wound healing

## Abstract

**Objectives:**

This randomized clinical trial evaluated the effects of three different antiseptic mouth rinses, chlorhexidine (CHX), CHX with anti-discoloration system (CHX ADS), and povidone-iodine (PVP-Iodine), on early wound healing, plaque formation, microbial load, inflammation biomarkers, and patient satisfaction after dental implant placement.

**Materials and methods:**

Sixty patients received one dental implant and were assigned by a computer-generated simple random allocation (random.org) to rinse with either CHX 0.2%, CHX ADS 0.2%, or PVP-Iodine 10% preoperatively and for 10 days postoperatively. No blinding was implemented. Primary outcome of the study was the assessment of the Early Wound Healing Index (EHI). Secondary outcomes included bacterial load of five periopathogens, levels of activated matrix metalloproteinase-8 (aMMP-8), plaque index (PI), and patient satisfaction. Statistical analysis employed Kruskal–Wallis and chi-square tests with significance set at *p* < 0.05.

**Results:**

No statistically significant differences were observed among the three groups in EHI scores, microbiological profiles, aMMP-8 levels, or PI at any time point. However, CHX ADS was rated significantly more favorably by patients regarding taste, dysgeusia, mucosal burning, and tooth discoloration (all *p* < 0.05). Interrater agreement on EHI was substantial (κ = 0.72).

**Conclusions:**

All tested mouth rinses demonstrated comparable efficacy in promoting early wound healing and infection control after implant surgery. CHX ADS showed superior patient acceptance, suggesting its potential as a preferred option based on subjective tolerability. Standardization of healing assessment indices is recommended for future studies to enhance comparability.

**Clinical relevance:**

While CHX, CHX ADS, and PVP-Iodine were equally effective for early peri-implant wound healing, CHX ADS showed better patient acceptance with fewer side effects (taste alteration, tooth discoloration), which is likely to improve compliance, reflecting enhanced tolerability rather than superior healing efficacy. This finding may guide clinicians in selecting antiseptic protocols that optimize both clinical outcomes and patient comfort and patient compliance.

## Introduction

Following dental implant insertion, antiseptic mouth rinses are commonly used for plaque control in the absence of mechanical cleaning to enhance postoperative wound healing and thus reduce the risk of infection and positively influence clinical outcomes [1, 2]. In particular, chlorhexidine (CHX) is one of the most frequently used antiseptics in dental medicine and is the gold standard for postoperative biofilm management [3]. The efficacy of CHX regarding the reduction of plaque accumulation and gingival inflammation following implant surgery has been confirmed in many studies [1, 4]. Nevertheless, several undesired side effects, such as staining of teeth and soft tissues, temporary taste alterations, allergies, and an increase in calculus formation have been reported[5]. Systematic reviews reported a large increase in tooth staining with CHX use (mean = 1.07; 95% CI: 0.80–1.34), and clinical trials have shown adverse events in patients using CHX mouth rinses, particularly at higher concentrations (0.2%) [6, 7]. One clinical study reported on 24% of tooth staining following rinsing with 0.12% CHX [8]. These side effects may compromise patient compliance and long-term acceptability, which led to the search for varied compositions and concentrations of CHX mouth rinses, as well as alternative antiseptics [1]. One example is the development of an anti-discoloration system (ADS) integrated into the CHX mouth rinse, which has shown positive effects on discoloration, food taste alterations, soft tissue irritation, and overall patient tolerance [4]. While one study showed the effectiveness of CHX with ADS equivalent to CHX without ADS in the reduction of soft tissue inflammation during the early healing phase [4] another study showed the opposite, thus not having a more pronounced effect on plaque index and gingival index than a placebo [9].

Another well-known antiseptic, broadly used as an antimicrobial agent in other medical disciplines, is polyvinylpyrrolidone-iodine (PVP-Iodine) [10, 11]. As a broad-spectrum antiseptic agent, it effectively eliminates bacteria, fungi, viruses, and other infection-causing pathogens [12–14]. It was shown that even after temporarily possibly leading to a decreased viability and impaired differentiation of cells, surviving cells recovered well, and no permanent resistance was developed [15]. PVP-iodine is primarily used for disinfecting minor wounds, skin lacerations, abrasions, and small-scale, superficial burns, and it is particularly suitable for application on mucous membranes and in the genital area [16]. Current evidence suggests that iodine's microbicidal effect is due to its interference with essential bacterial cellular functions and structures [12]. Specifically, it oxidizes critical components such as nucleotides and the fatty and amino acids within bacterial cell membranes, as well as cytosolic enzymes involved in the respiratory chain, thereby causing their denaturation and inactivation [17].

In dentistry, a mouth rinse based on PVP–iodine in combination with hydrogen peroxide (H_2_O_2_) has been used as adjunctive therapy to prevent the development of gingivitis [18]. Topically applied 0.1% PVP-Iodine showed to improve clinical outcomes of non-surgical periodontal therapy [19]. A recent study comparing patient reported outcome measures (PROMS) after post-operative rinsing with 0.5% PVP-Iodine versus rinsing with 0.12% CHX found significantly lower post-operative pain levels in the PVP-Iodine group. Seven days after implant placement, the results showed significantly less swelling in this group [8]. Even though the introduced antiseptics are frequently applied in dental medicine, literature mainly documents the utilization of PVP-Iodine mouth rinses as adjunctive therapy in non-surgical periodontal treatments [18–21]. The effectiveness of CHX on early wound healing, and the prevention of postoperative infections after dental implant placement has not been compared with CHX ADS and PVP-Iodine, to our knowledge. Further, only one study investigated on the PROMS and postoperative pain after the use of CHX and PVP-Iodine mouth rinses following implant insertion. This study applied different concentrations of the mouth rinse solutions, though. However, CHX with ADS was not included in this research [8]. It is of clinical importance to know, which antiseptic mouth rinse performs best in terms of efficacy on wound healing and prevention of infections, and additionally is tolerated most by patients, to ensure a good patient compliance during the treatment and healing period.

Therefore, the present randomized clinical trial aimed to assess the effects of three different antiseptic mouth rinses on early wound healing and prevention of postoperative infections at the peri-implant wound margins after implant placement. The null hypothesis was that there would be no differences in postoperative wound healing after using three different mouth rinses. Primary outcome of the study was the Early Wound Healing Index assessment (EHI). Further, the following parameters were assessed: plaque index, activated matrix metalloproteinase-8 (aMMP-8), the bacterial concentration of five marker organisms.

The conventional clinical examination is often insufficient to detect early peri-implant inflammatory responses, particularly in patients without active periodontal disease. Activated matrix metalloproteinase-8 (aMMP-8), a neutrophil-derived collagenase involved in extracellular matrix degradation, has been established as a sensitive and specific biomarker for active periodontal and peri-implant inflammation. Elevated aMMP-8 levels in peri-implant crevicular fluid have been associated with early tissue responses and may reflect the biological activity of wound healing or early inflammation [22]. In this randomized controlled clinical trial, aMMP-8 levels were monitored to evaluate the effect of different mouth rinses on peri-implant inflammatory response during the early healing phase.

## Materials and methods

The following antiseptic mouth rinses were investigated, focusing on the peri-implant healing pattern in patients receiving dental implants:

- **Chlorhexamed® 0.2%** (CHX) (GlaxoSmithKline Consumer Healthcare GmbH, Brentford, England)
- **Curasept® 0.2%** (CHX ADS) (Curaden, Kriens, Switzerland)
- **Betadine® Povidone-Iodine 10%** (PVP-I) (Mundipharma, Basel, Switzerland)

Chlorhexamed 0.2% (CHX) served as the control group, while Curasept® 0.2% (CHX ADS) and Betadine® povidone-iodine 10% (PVP-Iodine) were the two test groups.

The study protocol was approved prior to the initiation of the study by the responsible ethics committee and the Swiss federal supervisory authority, Swissethics (BASEC No. 2016–00721). The study was performed according to the Declaration of Helsinki on medical protocol and ethics and in compliance with CONSORT guidelines for randomized trials. All patients were informed about the surgical procedures and the purpose of applying mouth rinses before and after the intervention. Each participant provided oral and written informed consent after having had the opportunity for questions.

Strict inclusion criteria were applied to avoid confounding factors that could have influenced the effect of mouth rinsing solutions.

The following exclusion criteria were defined:

- Simultaneous bone augmentation requiring antibiotic prophylaxis
- Osteoporosis treatment with bisphosphonates necessitating concomitant intake of antibiotics
- Pregnancy
- Thyroid disorders requiring medication
- Uncontrolled diabetes mellitus
- Autoimmune diseases affecting wound healing
- Allergies to CHX or PVP-Iodine
- Heavy smoking (more than 19 cigarettes per day)

**Fig. 1:**
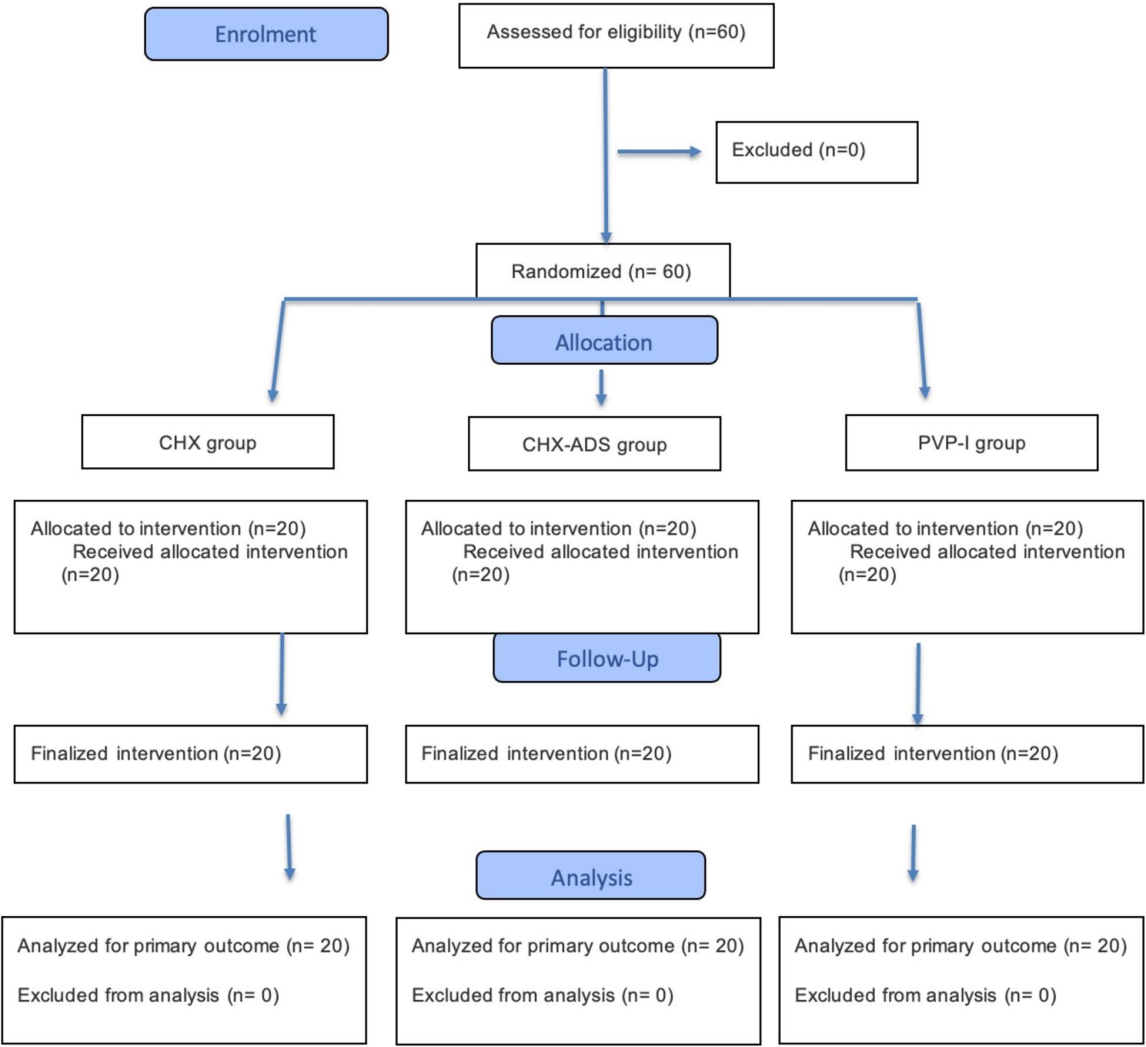
CONSORT Flow Diagram: Flow Diagram of the progress through the phases of enrolment, intervention allocation, follow-up and data analysis of the 3 groups (CHX, CHX ADS, and PVP-I) of this RCT

Sixty participants who had received at least one dental implant (OsseoSpeed™ EV, Astra Tech Implant System, Dentsply Implants) in the maxilla (n = 35) or mandible (n = 25) in anterior or posterior position were included in the study (CONSORT Flow Diagram (Fig. 1). The study was conducted in a private dental office, and surgeries and all postoperative follow-up examinations, including the assessment of patient-reported outcome measures (PROMs) were performed by one experienced implantologist (ME), who placed at least 200 implants per year. Randomization was performed using a computer-generated randomization list (random.org), assigning a 20 patients to each group (Fig. 1). No blinding was performed. Patients were instructed to rinse with one of the three randomly assigned antiseptic mouth rinses directly before surgery and for 10 consecutive days postoperatively, three times per day, for one minute each. To ensure adherence to the protocol, patients were thoroughly advised how to rinse, and the clinician explained the rationale for this treatment.

### Surgical treatment

In all 60 patients, a mid-crestal incision, partially with mesial release incision, was performed. After mucosal flap preparation, the implant bed was prepared using drills manufactured by Dentsply Implants (Dentsply Sirona, Bensheim, Germany) according to the manufacturer's specifications. The designated implant was then inserted into the prepared site. In case indicated, simultaneous bone augmentation was performed with autologous bone chips, bovine xenogeneic bone particles (BioOss, Geistlich Pharma, Wolhusen, Switzerland) and a resorbable collagen membrane (BioGide, Geistlich Pharma, Wolhusen, Switzerland) to cover the added bone particles. Implants were submerged and primary flap closure was achieved with single interrupted and horizontal mattress sutures (Supramid, size 3/0, Braun, Melsungen, Germany). Release incisions were closed using Supramid size 5/0 (Supramid, size 5/0, Braun, Melsungen, Germany).

### Effect on wound healing

#### Wound healing index

The surgeon photographically documented the postoperative wound healing process at five and ten days after surgery Table 1. The EHI was separately evaluated at a later stage by two independent examiners (PSA, PSH) based on this documentation using the criteria of the Early Wound-Healing Index (EHI) [23] on a five-point scale (Table 2). The photographs were blinded, so that no attribution to the corresponding mouth rinse group or patient could be made by the evaluators. This way, the evaluators were blinded when assessing the EHI ratings.

**Table 1:** Distribution of smokers and gender to the three different mouth rinses CHX/CHX ADS/PVP-Iodine

**Category**	CHX	CHX ADS	PVP-Iodine
Smoker	13	12	12
Female	6	11	10
Male	14	9	10

**Table 2:** Early Wound Healing Index (EHI) (Wachtel, Schenk et al. 2003)

1	Complete flap closure	No fibrin line in the interproximal area
2	Complete flap closure	Slight fibrin line in the interproximal area
3	Complete flap closure	Fibrin coagulum in the interproximal area
4	Incomplete flap closure	Partial necrosis of the interproximal tissue
5	Incomplete flap closure	Complete necrosis of the interproximal tissue

#### Patient-reported outcome measures (PROMs)

After 10 days, the patients were asked to report their perception of the surgical outcome by marking a point on a Visual Analogue Scale (VAS) according to their satisfaction, ranging from 0 (very satisfied) to 10 (not satisfied at all). The VAS was reported for seven different criteria (Table 3).

**Table 3:** Criteria for the Visual Analogue Scales (VAS) for Patient-reported outcome measures (PROMs)

	Criteria	Value 0 on scale	Value 10 on scale
1	Satisfaction with wound healing	completely	not at all
	Taste of the Mouth rinse	angenehm	very unpleasant
	Dysgeusia (Disorder of the sense of taste)	no	strong
	Tooth discoloration	no	strong
	Burning of the oral mucosa	no	strong
	Postoperative pain	no	intolerable
	Amount of painkillers	none	many

### Antiseptic effect

The preoperative status, the antiseptic effect of the mouth rinses, and the postoperative wound healing were assessed. For assessing the antiseptic effect of the tested mouth rinsing solutions, the following three methods were performed:

1. Microbiological evaluation

The microbiological evaluation was performed using the molecular-biological detection method micro-IDent®Plus (Heicodent, Wolfhausen, Switzerland), combining polymerase chain reaction (PCR) with DNA strip technology. The microbial analysis facilitates the evidence-based selection of optimally effective medications. It enables the detection of five periodontal pathogenic bacterial complexes with 11 subgroups, namely *Aggregatibacter actinomycetemcomitans*, *Prevotella intermedia, Tannerella forsythia,* Porphyromonas gingivalis, and *Treponema denticola* [24].

For the micro-IDent®Plus test, samples of the intraoral microflora of the surgical site were taken with a sterile curette from the back of the tongue and the saliva pre-operatively and 5 and 10 days postoperatively. Additionally, samples were collected postoperatively from 3 predefined sutures. Test results were categorized into five categories according to the bacterial concentration. The findings were directly compared between the three mouth rinse solutions.

2) Biomarker assessment

For the Biomarker assessment, the quantitative biomarker PerioMarker® (Hager und Werken, Duisburg, Deutschland) was used for detecting the presence of activated matrix metalloproteinases-8 (aMMP-8) based on a standardized enzyme-linked immunosorbent assay (ELISA) test procedure. A collection strip was inserted into the early sulcus of the placed implant to collect samples of the sulcular fluid. Subsequently, quantitative analysis was performed in the laboratory using the sample eluate. All aMMP-8 measurements were reported in ng/ml. A threshold value of 8 ng/ml aMMP-8 in the sulcular fluid was set to differentiate between clinically healthy gingiva/mucosa and chronic periodontitis/peri-implantitis.

3) Plaque determination

The plaque index (PI), according to *O’Leary* [25], was used to assess the biofilm accumulation at the two neighboring teeth, if present. The PI was calculated using the formula: PI = Number of plaque-covered surfaces/total number of tooth surfaces × 100. The PI value demonstrates the "contamination" of the oral cavity in percent.

### Statistical analysis

The statistical analysis of the collected data and plots were generated using the statistical software R (© The R Foundation) including ggplot2 [26].

To calculate the minimum number of participants required in each group, a sample size calculation was performed.

With a power of 80%, a significance level of 5%, and an expected intergroup difference of 0.5 (with a standard deviation of 0.5) for EHI, the minimum required sample size was determined to be 16 participants per group. To account for potential loss to follow-up, the decision was made to enroll 20 participants in each group.

In descriptive analyses, the mean with standard deviation (SD), the median, and the interquartile range (IQR) were calculated to describe the data dispersion. Boxplots displayed the median, the interquartile range, and extreme values or outliers of individual variables.

A non-parametric chi-square test was used to determine whether the three mouth rinses exhibited statistically significant differences concerning nominal parameters (cases of aMMP-8 > 8 ng/ml). A non-parametric Kruskal–Wallis test was also employed to assess whether the three mouth rinses showed statistically significant differences in metric parameters at defined time points. No additional correction for multiple VAS outcomes was applied, as these analyses were exploratory. P-values < 0.05 were considered statistically significant; conversely, p-values ≥ 0.05 indicated no differences between the mouth rinses regarding a target variable. Interrater reliability was evaluated using weighted Cohen's Kappa with quadratic weighting, which is appropriate for ordinal data.

## Results

Sixty patients with a mean age of 54.6 years (29.0–80.3 years) received 60 dental implants. Thirty-three patients (55.0%) were male, and 27 (45.0%) were female. The proportion of both genders and smokers in the three different groups is shown in Table 1. Overall, 26.7% (N = 16) of the patients were smokers, with an average cigarette consumption of 10.9 cigarettes per day, and 35.0% (N = 21) were former smokers. In 73.3% of patients, simultaneous bone augmentation was performed during implant placement, notably with no intake of antibiotics.

### Primary outcome: Wound healing index—EHI

**Fig. 2:**
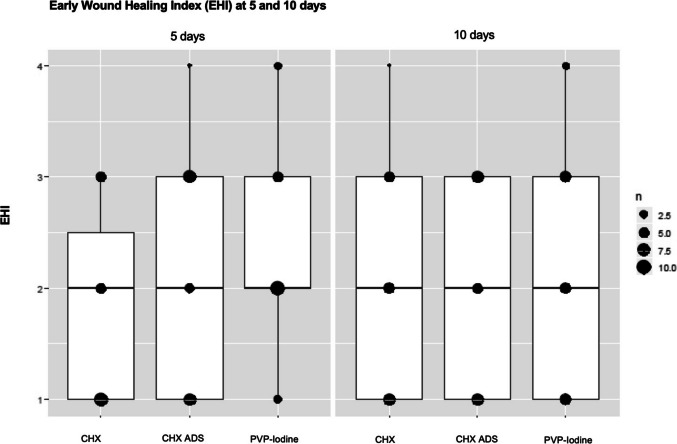
Early wound healing Index (EHI) with scores from 1–4 at 5 and 10 days. Boxplots showing the relationship between the mouth rinses and the EHI at different time points

The results for the primary outcome in the overall cohort when examining the relationship between the mouth rinses and the EHI at the three different assessment time points is given in Fig. 2. A median EHI of 2.0 (IQR ranging from 1.0–2.0) was found for all three mouth rinses at five and ten days postoperatively. The Kruskal–Wallis test revealed no significant differences in EHI between the three mouth rinses at any follow-up time points (p = 0.22 at 5 days postoperatively and p = 0.79 at 10 days postoperatively) (Table 4). The interrater reliability analysis yielded a Kappa value of 0.72, which reflects substantial agreement between the two raters.

**Table 4:** Summary of main outcome values by mouth rinse group, with measures of dispersion and p-values

Outcome & Time Point	**CHX** Median [IQR] Mean ± SD [95% CI (n = 20)]	**CHX ADS** Median [IQR] Mean ± SD [95% CI (n = 20)]	**PVP-I** Median [IQR] Mean ± SD [95% CI (n = 20)]	**p-value**
EHI Day 5	2.0 [1.5] 1.789 ± 0.855 [1.39, 2.19]	2.0 [2.0] 2.150 ± 0.988 [1.69, 2.61]	2.0 [1.0] 2.300 ± 0.865 [1.90, 2.70]	0.22
EHI Day 10	2.0 [2.0] 2.000 ± 0.943 [1.56, 2.44]	2.0 [2.0] 2.000 ± 0.88 [1.59, 2.41]	2.0 [2.0] 2.200 ± 1.005 [1.73, 2.67]	0.79
VAS – Taste	4.0 [2.25] 4.08 ± 2.17	2.0 [1.25] 1.95 ± 1.53	5.0 [3.00] 6.17 ± 2.64	< 0.001
VAS – Dysgeusia	5.0 [5.25] 4.95 ± 3.05	0.0 [0.13] 0.50 ± 1.27	2.00 [4.63] 2.48 ± 2.68	< 0.001
VAS – Tooth staining	1.0 [3.25] 2.10 ± 2.32	0.0 [1.00] 0.53 ± 1.06	1.0 [3.13] 1.73 ± 1.83	0.019
VAS – Burning oral mucosa	5.0 [1.38] 4.55 ± 2.41	0.0 [1.25] 1.12 ± 1.93	0.5 [2.25] 1.65 ± 2.18	< 0.001
aMMP-8 Day 0 (ng/ml)	10.20 [6.70] 12.70 ± 8.99 [8.49, 16.91]	11.10 [11.83] 13.89 ± 8.31 [10.00, 17.78]	11.35 [11.05] 14.89 ± 10.33 [10.06, 19.72]	0.762
aMMP-8 Day 10 (ng/ml)	9.75 [6.75] 11.71 ± 9.74 [7.15, 16.27]	9.55 [6.45] 12.83 ± 9.26 [8.50, 17.16]	13.25 [8.12] 15.70 ± 12.39 [9.90, 21.50]	0.189
Plaque Index Day 0 (%)	59.6 [18.7] 56.3 ± 15.4	50.1 [22.1] 51.2 ± 16.6	62.3 [25.0] 57.3 ± 17.2	0.511
Plaque Index Day 5 (%)	43.3 [13.3] 43.2 ± 12.3	37.0 [17.5] 40.0 ± 17.3	41.8 [18.3] 42.3 ± 13.3	0.565
Plaque Index Day 10 (%)	36.8 [11.1] 37.1 ± 13.3	40.5 [21.1] 42.4 ± 13.8	34.2 [23.5] 36.0 ± 14.7	0.281

**Abbreviations:** CHX = chlorhexidine; CHX ADS = chlorhexidine with anti-discoloration system; PVP-I = povidone-iodine; EHI = early wound healing index; VAS = visual analogue scale; aMMP-8 = active matrix metalloproteinase-8; PI = plaque index; IQR = interquartile range; SD = standard deviation

### Patient perception

**Fig. 3:**
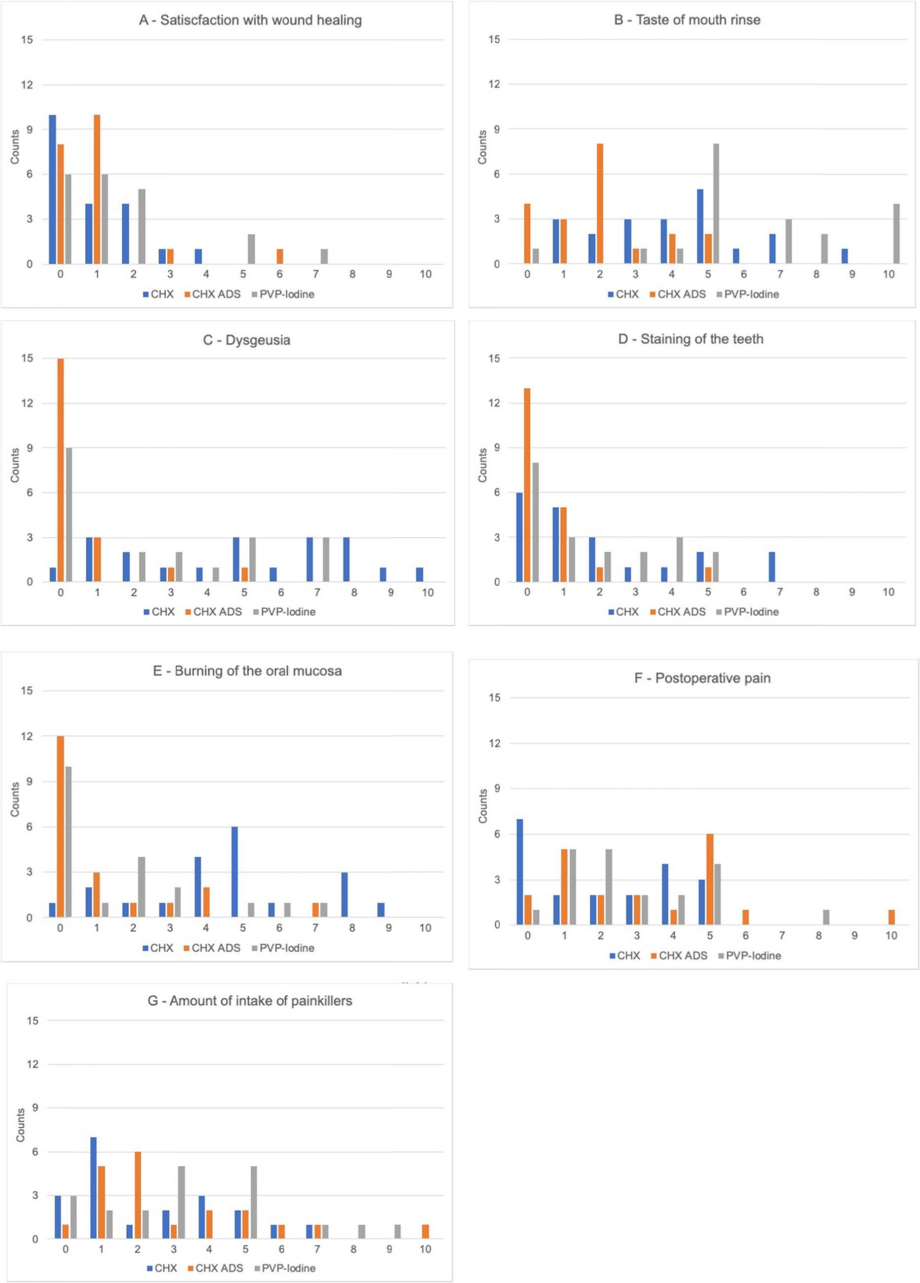
**A-G:** Patient reported outcome measures: VAS Scores for the VAS Criteria A—E: Satisfaction with wound healing (A), Taste of mouth rinse (B), Dysgeusia (C), Staining of the teeth (D), Burning of the oral mucaosa (E), Postoperative Pain (F), Amount of intake of painkillers (G)

**Fig. 4:**
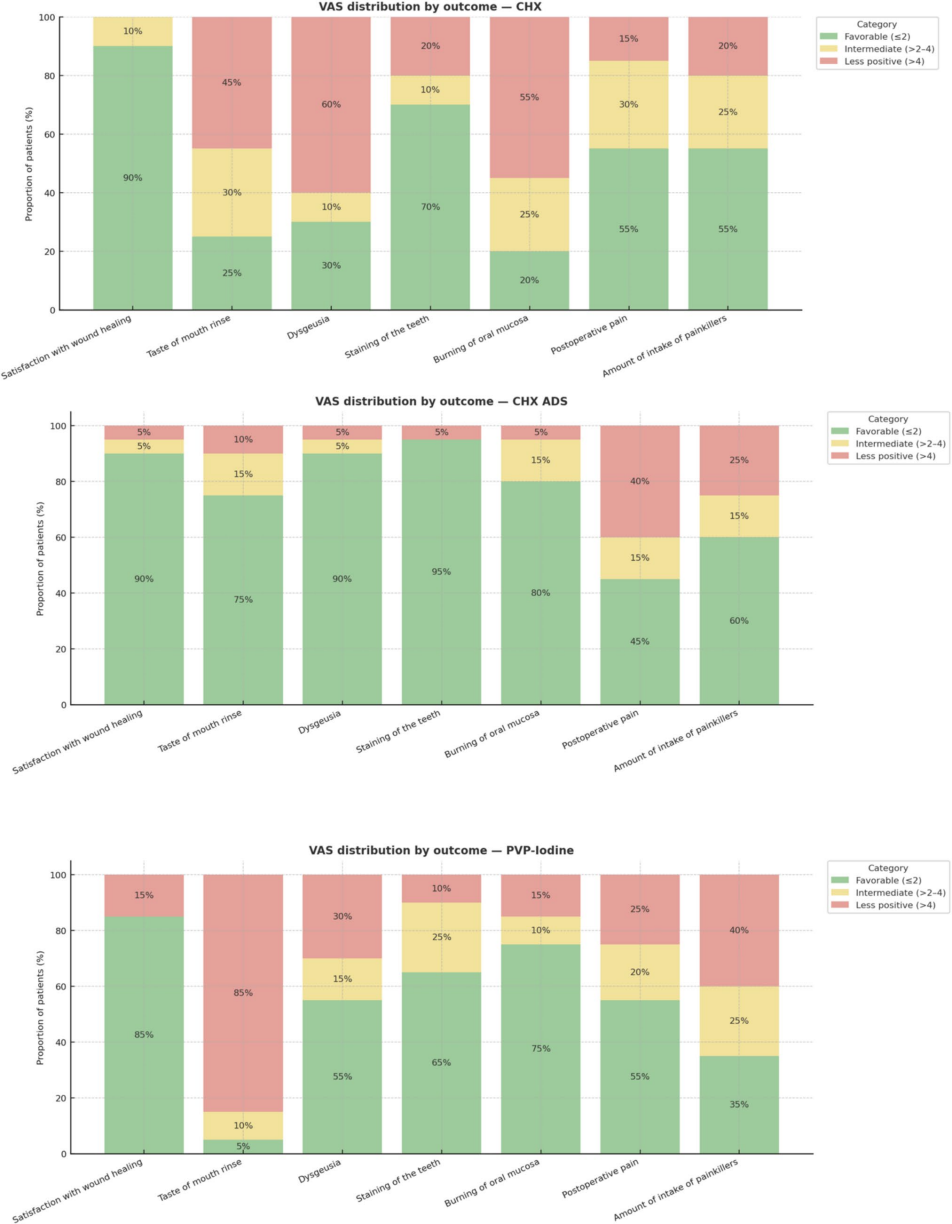
**A-C:** Frequency distribution of patients rating the A: CHX; B: CHX ADS and C: PVP-Iodine mouth rinses favorably or less positively. Thresholds: Favorable: VAS scores ≤ 2; Intermediate: VAS scores > 2 to 4; Less positive: VAS scores > 4. Depicted are all seven VAS criteria

The mean values for the seven VAS criteria showed that all patients were generally satisfied with the healing process (Figs. 3 A-G). However, inter-group differences between the three types of mouth rinses revealed significantly lower (better) VAS scores for the CHX ADS mouth rinse in four of the seven criteria (Fig. 4 A-C). The taste of the mouth rinse CHX ADS (mean = 2.0) was rated significantly better (p < 0.001) than PVP-iodine (mean = 6.2) and CHX (mean = 4.1) (Fig. 3B; Table 1). Regarding dysgeusia following the use of a mouth rinse, CHX ADS (mean = 0.5) was also rated significantly better; p < 0.001) than CHX (mean = 5.0) and PVP-Iodine (mean = 2.5) by the patients (Fig. 3C; Table 1). CHX ADS led to significantly less tooth staining (mean = 0.5) according to the patient VAS rating compared with CHX (mean = 2.1) and PVP-Iodine (mean = 1.7) with p = 0.019 (Fig. 3D; Table 1). Finally, patients perceived less burning of the oral mucosa after having rinsed with CHX ADS (mean = 1.1) than with CHX (mean = 4.6) and PVP-Iodine (mean = 1.7) with p < 0.001(Fig. 3E; Table 1).

### Microbiological evaluation

**Fig. 5:**
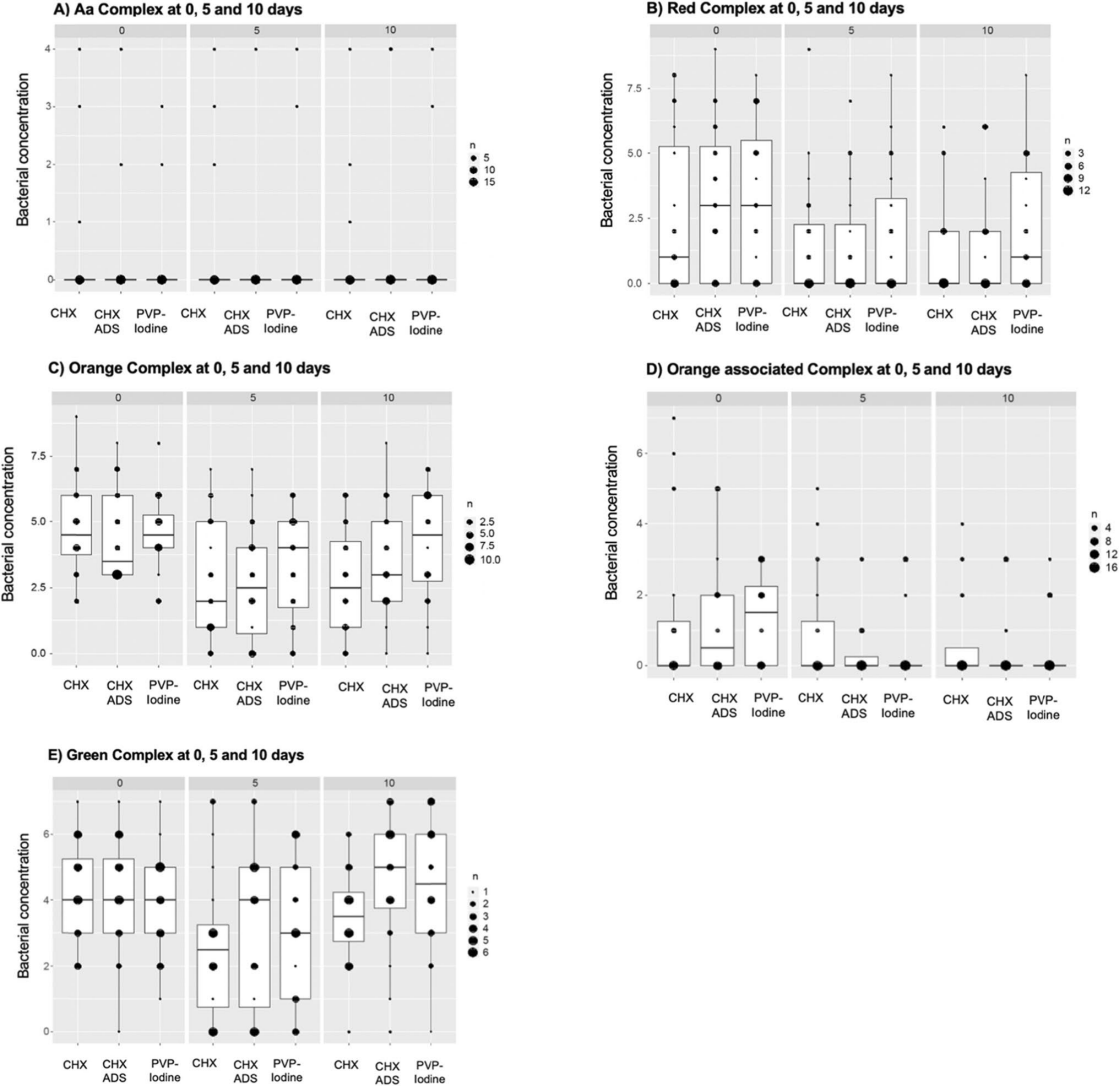
**A-E:** Results of the micro-IDent®Plus test (Hain Lifescience GmbH, Nehren, Germany). Boxplots showing the relationship between the mouth rinses and the bacterial concentration for the five bacterial complexes (A: Aa, B: red, C: orange, D: orange associated, and E: green complex) at the three different time points (0, 5, and 10 days) Biomarker assessment: ammp8 levels at 0 and 10 days. Boxplots showing the relationship between the mouth rinses and the aMMP8 values

The relationship between the mouth rinses and the bacterial concentration for the five bacterial complexes at the three different time points is shown in Fig. 5. The Kruskal–Wallis test showed no significant differences in bacterial concentration among the three mouth rinses for all five micro-IDent complexes at any time (p > 0.05 for all complexes at days 0, 5 and 10).

### Biomarker assessment

**Fig. 6:**
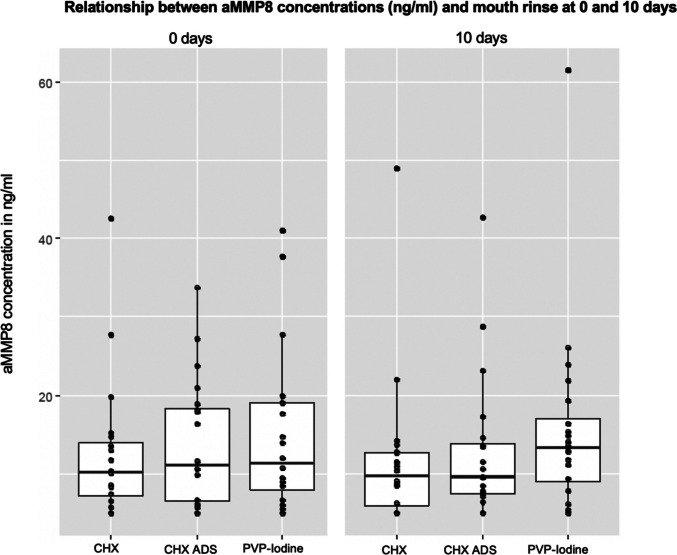
Results of the Biomarker assessment: ammp8 concentrations (ng/ml) at 0 and 10 days. Boxplots showing the relationship between the mouth rinses and the aMMP8 values

When assessing the relationship between the mouth rinses and the biomarker aMMP-8, the median aMMP-8 value on the day of surgery was lowest in patients using CHX at 10.2 (IQR = 6.7) and highest in patients using PVP-Iodine at 11.4 (IQR = 11.1). The median aMMP-8 value ten days postoperatively was lowest in patients using CHX ADS at 9.6 (IQR = 6.5) and highest in patients using PVP-Iodine at 13.3 (IQR = 8.1) (Fig. 6). The Kruskal–Wallis test revealed, however, no significant differences in aMMP-8 levels between the three mouth rinses at both measurement time points (p = 0.762 at the day of surgery, and p = 0.189 at 10 days postoperatively) (Table 4). When considering only acute clinical tissue breakdown cases with an aMMP-8 threshold > 8 ng/ml in sulcus fluid, the distribution across the three mouth rinses at the two measurement time points was also not statistically significantly different (p ≥ 0.05).

### Plaque assessment

**Fig. 7:**
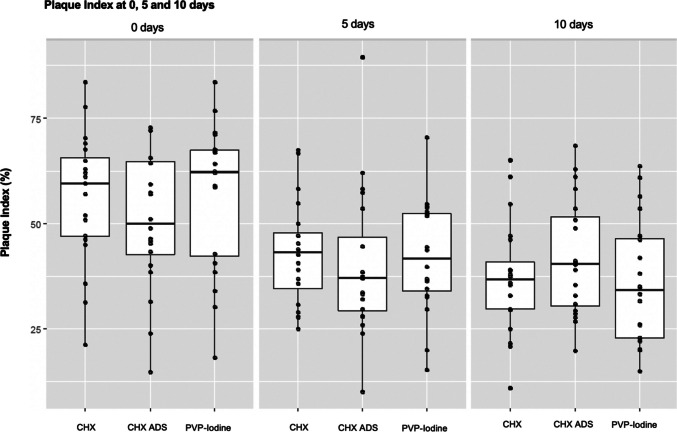
Plaque Index at 0, 5, and 10 days for the three mouth rinses. Boxplots showing the relationship between the mouth rinses and the Plaque Index

The mean PI on the day of surgery was highest in patients using PVP-Iodine at 57.3% ± 17.2, while 10 days postoperatively, the lowest mean value was observed in the same group at 36.1% ± 14.7 (Fig. 7). The Kruskal–Wallis test did not detect any significant difference in the PI among the three mouth rinses at any of the three time points (at 0 days: p = 0.511; at 5 days: p = 0.565 and at 10 days: p = 0.281). The median PI 10 days after surgery was lowest in patients using PVP-Iodine at 34.2% (IQR = 24.0) (Table 4).

## Discussion

The present study aimed to evaluate the effects of three different mouth rinse solutions on early wound healing after dental implant placement. The results of this study confirmed the hypothesis that there are no differences in the antiseptic effects, early wound healing, and prevention of postoperative infections after using the three assessed mouth rinses following implant surgery. This included the EHI, the microbiological evaluation, the biomarker assessment with analysis of aMMP-8 levels, and plaque indices. However, patient-reported outcome measures (PROMS) revealed a significant preference for CHX ADS mouth rinse in more than half of the analyzed criteria.

The EHI was selected as the primary outcome and assessed independently by two clinical experts following the criteria of Wachtel et al. [23], demonstrating substantial inter-rater agreement. The EHI offers a standardized and reproducible method to evaluate soft tissue healing in the critical early phase after surgery [27], making it a valuable tool for postsurgical monitoring and ensuring wound stability, which is essential for long-term treatment success [28]. Because bone augmentation was performed in a defect-oriented manner and not uniformly across all patients, variations in augmentation volume may have influenced flap adaptation and thereby affected EHI scores. Nevertheless, a tension-free closure was ensured in all cases, which minimized the potential impact of this variable on early wound healing outcomes. High scores enable clinicians to identify potential healing disturbances at an early stage and intervene before serious complications arise. The inter-rater reliability observed in this study was comparable to that reported in previous investigations, further supporting the robustness of the EHI for early wound healing assessment [27].

The superior VAS ratings for CHX ADS likely reflect the anti-discoloration system, which reduces staining, taste alterations, and soft tissue irritation [4]. These differences, however, should not be viewed as evidence of superior wound healing efficacy, as all rinses showed comparable outcomes. Rather, they underscore the role of patient-centered considerations in the choice of antiseptic. The enrolment in the study guaranteed a close patient monitoring during this critical early healing phase. By minimizing side effects, CHX ADS may enhance compliance with postsurgical protocols and thereby support favorable early healing, particularly in patients not subject to such close clinical monitoring.

The plaque index according to *O’Lear*y [25] is a standard procedure in dental medicine to evaluate the presence and amount of biofilm on teeth, and it allows to draw conclusions about the oral hygiene level of patients. In a systematic review including 11 publications analyzing the efficacy of CHX rinses after periodontal or implant therapy, CHX significantly reduced plaque accumulation and bleeding compared to placebo controls [1]. The authors concluded that CHX mouth rinsing contributes to minimizing biofilm accumulation and alleviating gingival inflammation in the postoperative period. These findings align with the present study's results, which revealed a reduction of the PI in the CHX group from the day of surgery to 10 days postoperatively.

Both the direct molecular detection method micro-IDent®plus and the indirect test for inflammatory periodontal tissue breakdown, via quantitative determination of activated matrix metalloproteinase-8 using PerioMarker®, are well-established methods for the detection of periodontitis and peri-implantitis [29–31]. Active matrix metalloproteinase-8 has emerged as a promising biomarker for monitoring peri-implant tissue health and early wound healing. As a predominant collagenase in periodontal and peri-implant inflammation, elevated aMMP-8 levels reflect collagen degradation and are strongly associated with the onset and progression of peri-implant diseases. Its use in implant dentistry therefore offers the advantage of providing an objective, chairside-accessible measure of soft tissue response, complementing traditional clinical indices that may be more subjective or less sensitive to early changes. Including additional biomarkers such as interleukin-1β (IL-1β) and tumor necrosis factor-alpha (TNF-α) in future studies could enable a more comprehensive monitoring of early wound healing following implant placement. These cytokines play central roles in modulating inflammatory responses, bone metabolism, and tissue repair. Their combined evaluation alongside aMMP-8 could allow for a more nuanced characterization of the healing trajectory, identification of patients at risk for impaired healing, and possibly the development of personalized postoperative monitoring strategies [31].

As PVP-Iodine presents a broad spectrum of activity, low toxicity, and only a few contraindications, it is suggested as an antiseptic mouth rinse to enhance wound healing after periodontal therapy or surgery [10, 11, 19]. However, PVP-Iodine did not outperform the CHX-based mouth rinse in the present study. The antiseptic effect of PVP-Iodine was similar to the effectiveness of CHX and CHX ADS regarding the microbiological evaluation, biomarker assessment, the presence of plaque, and wound healing. Moreover, PVP-Iodine may be accompanied by other risks, such as cytotoxicity and reduction of cell proliferation of fibroblasts and osteoblasts, depending on the concentration applied [15, 32, 33]. Hence, caution is advised in cases of alveolar bone exposure and tissue hypersensitivity due to the low pH value of PVP-Iodine.

Although a sample size calculation was performed prior to study initiation, the lack of significant differences in antiseptic effects between the three mouth rinses may be attributed to the limited sample size (n = 20), which may have reduced the ability to detect clinically relevant effects. To assess the likelihood of detecting an existing difference between the groups, a post-hoc power analysis was conducted based on the present data. This analysis confirmed low statistical power (5–30%), which is typical for datasets without significant findings, suggesting that substantially larger samples (estimated > 100 participants) would be required to detect a clinically meaningful difference in EHI of 1.0. Nonetheless, the standardized surgical protocol performed by a single clinician likely minimized variability across groups, supporting the internal consistency of the results.

Even though a negative control could have provided baseline data on natural healing, it was not included as rinsing with CHX is the established gold standard after implant placement, making a placebo control ethically and clinically unjustifiable. Instead, the study focused on comparing three relevant antiseptic rinses, thereby ensuring the results are directly applicable to daily clinical practice. During healing, adherence to the rinsing protocol was not objectively monitored to mirror routine practice; however, all patients were informed about the rationale for antiseptic rinsing to emphasize the importance of compliance. Because adherence was not objectively verified, any non-compliance may have introduced uncontrolled bias, potentially affecting the observed effects. Moreover, the analysis was limited to between-group comparisons; no within-group analyses were performed. Assessing changes within each arm—such as plaque index from day 0 to day 10—could illuminate treatment-specific outcomes and should be included in future studies. Also, it would have been informative to test whether any correlation exists between biological (aMMP8 values) and clinical (EHI ratings) parameters. This could be addressed in future study designs. Correlations between the biomarker (aMMP-8) and the clinical endpoint (EHI) were not assessed in this study, as the study was not powered for detecting such associations. Future studies with larger samples should test biomarker–clinical correlations (e.g., Spearman rank) to clarify whether changes in aMMP-8 predict clinical healing.

A further limitation of this study is the absence of blinding, which may have led to a response bias of patients concerning the PROMs and a bias of the evaluator clinical outcomes. Knowing their allocated rinse could have influenced patients rating the experienced side effects (taste, tooth staining, burning). However, this open-label design reflects real-world conditions in implant aftercare, where blinding of rinsing agents is rarely feasible. Future trials should, where feasible, use double-blind designs and complement PROMs with objective measures to mitigate these biases. One objective approach could be the measurement of the degree of staining by means of spectrophotometry. Nevertheless, the evaluation of the primary outcome measure, the EHI, was performed blinded, so that no attribution of the clinical pictures to the study groups or patients was possible. A limitation of the EHI is its restricted comparability with other studies, as diverse wound-healing indices and parameters are reported in the literature, including membrane exposures, incision margins, dehiscence, abscess formation, edema, suppuration, swelling, scar tissue, pain, and allergic reactions [34, 35]. This heterogeneity hampers meaningful comparison and interpretation of outcomes, and no consensus has yet been reached on the most appropriate scale or time frame for wound healing assessment.

This study is also limited by the VAS questionnaire used. Although the items to be evaluated were clearly presented by focusing on the specific aspect of concern (sometimes one word only), the questionnaire had not been previously validated. Therefore, no conclusions can be drawn regarding its reliability or the consistency of the patients’ responses. Perceptions such as «pain» or «satisfaction with healing» are multifactorial [36, 37] and patients may experience sensory impressions such as «dysgeusia» in very different ways. Nevertheless, the results contribute to a better understanding of potential differences arising from the use of different antiseptic mouth rinses and may provide a useful basis for the design and validation of future PROM instruments.

Although not registered prospectively, the study adhered to CONSORT guidelines and the dataset was complete and transparently reported. Future studies should be registered. Further, future studies should include an increased sample size to improve the significance of the study, as well as longer follow-up periods (> 10 days) to monitor the progression of wound healing. Additional groups could be included, such as patients with certain conditions, for example periodontitis, or heavy smokers, which would help to better understand the tissue responses and post-operative care needed for these patient groups. Multi-marker approaches (including aMMP8, TNF-α, IL-1β) as described above, may ultimately enhance diagnostic accuracy and improve long-term outcomes in implant therapy.

## Conclusions

Among the tested mouth rinse solutions no statistically significant differences in respect to antiseptic effects, early wound healing, and prevention of postoperative infection were observed. Statistically significant differences were observed only in the subjective VAS ratings. Consequently, the choice of antiseptic mouth rinse could have an impact on the patient compliance during home follow-up care.

Considering the variety of indices used to assess the postoperative tissue healing after dental implant insertion, a consensus on the standardization of applied indices would be desirable to enhance the comparability of research findings.

## Data Availability

The data that support the findings of this study are not openly available due to reasons of sensitivity and are available from the corresponding author upon reasonable request.

## References

[1] Solderer A, Kaufmann M, Hofer D, Wiedemeier D, Attin T, Schmidlin PR (2019) Efficacy of chlorhexidine rinses after periodontal or implant surgery: a systematic review. Clin Oral Investig 23(1):21–32. 10.1007/s00784-018-2761-y 30535817 10.1007/s00784-018-2761-y

[2] Poppolo Deus F, Ouanounou A (2022) Chlorhexidine in dentistry: pharmacology, uses, and adverse effects. Int Dent J 72(3):269–277. 10.1016/j.identj.2022.01.005 35287956 10.1016/j.identj.2022.01.005 PMC9275362

[3] Loe H, Schiott CR (1970) The effect of mouthrinses and topical application of chlorhexidine on the development of dental plaque and gingivitis in man. J Periodontal Res 5(2):79–83. 10.1111/j.1600-0765.1970.tb00696.x 4254172 10.1111/j.1600-0765.1970.tb00696.x

[4] Cortellini P, Labriola A, Zambelli R, Prato GP, Nieri M, Tonetti MS (2008) Chlorhexidine with an anti discoloration system after periodontal flap surgery: a cross-over, randomized, triple-blind clinical trial. J Clin Periodontol 35(7):614–620. 10.1111/j.1600-051X.2008.01238.x 18422695 10.1111/j.1600-051X.2008.01238.x

[5] Sanz M, Vallcorba N, Fabregues S, Müller I, Herkströter F (1994) The effect of a dentifrice containing chlorhexidine and zinc on plaque, gingivitis, calculus and tooth staining. J Clin Periodontol 21(6):431–437. 10.1111/j.1600-051x.1994.tb00741.x 8089246 10.1111/j.1600-051x.1994.tb00741.x

[6] James P, Worthington HV, Parnell C, Harding M, Lamont T, Cheung A et al (2017) Chlorhexidine mouthrinse as an adjunctive treatment for gingival health. Cochrane Database Syst Rev 3(3):Cd008676. 10.1002/14651858.CD008676.pub2 28362061 10.1002/14651858.CD008676.pub2 PMC6464488

[7] Van Strydonck DA, Slot DE, der Van Velden U, der Van Weijden F (2012) Effect of a chlorhexidine mouthrinse on plaque, gingival inflammation and staining in gingivitis patients: a systematic review. J Clin Periodontol 39(11):1042–1055. 10.1111/j.1600-051X.2012.01883.x 22957711 10.1111/j.1600-051X.2012.01883.x

[8] Chen J, Chen Z, Yan Q, Yin R, Feng Y, Wang L (2025) Patient-reported outcome measures comparing povidone iodine rinse and chlorhexidine rinse for dental implant therapy: a randomized controlled trial. BMC Oral Health 25(1):1233. 10.1186/s12903-025-06560-8 40702480 10.1186/s12903-025-06560-8 PMC12285018

[9] Varoni EM, Gargano M, Ludwig N, Lodi G, Sardella A, Carrassi A (2017) Efficacy of an anti-discoloration system (ADS) in a 0.12% chlorhexidine mouthwash: a triple blind, randomized clinical trial. Am J Dent 30(5):235–242 29178725

[10] Barreto R, Barrois B, Lambert J, Malhotra-Kumar S, Santos-Fernandes V, Monstrey S (2020) Addressing the challenges in antisepsis: focus on povidone iodine. Int J Antimicrob Agents 56(3):106064. 10.1016/j.ijantimicag.2020.106064 32599228 10.1016/j.ijantimicag.2020.106064

[11] Svellenti L, Karacic J, Herzog J, Tanner M, Sahrmann P (2024) Effects of rinsing with Povidone-Iodine during step II periodontal therapy: a systematic review and meta-analysis. J Clin Med. 10.3390/jcm13072111 38610876 10.3390/jcm13072111 PMC11012979

[12] Kawana R, Kitamura T, Nakagomi O, Matsumoto I, Arita M, Yoshihara N et al (1997) Inactivation of human viruses by povidone-iodine in comparison with other antiseptics. Dermatology 195(Suppl 2):29–35. 10.1159/000246027 9403252 10.1159/000246027

[13] Wutzler P, Sauerbrei A, Klocking R, Brogmann B, Reimer K (2002) Virucidal activity and cytotoxicity of the liposomal formulation of povidone-iodine. Antiviral Res 54(2):89–97. 10.1016/s0166-3542(01)00213-3 12062394 10.1016/s0166-3542(01)00213-3

[14] Schreier H, Erdos G, Reimer K, Konig B, Konig W, Fleischer W (1997) Molecular effects of povidone-iodine on relevant microorganisms: an electron-microscopic and biochemical study. Dermatology 195(Suppl 2):111–116. 10.1159/000246043 9403268 10.1159/000246043

[15] Schmidlin PR, Imfeld T, Sahrmann P, Tchouboukov A, Weber FE (2009) Effect of short-time povidone-iodine application on osteoblast proliferation and differentiation. Open Dent J 3:208–212. 10.2174/1874210600903010208 19915721 10.2174/1874210600903010208 PMC2776307

[16] Pollack W, Iny O (1985) A physico-chemical study of PVP-I solutions leading to the reformulation of “Betadine” preparations (5% PVP-I). J Hosp Infect 6:25–32. 10.1016/s0195-6701(85)80042-6 2860172 10.1016/s0195-6701(85)80042-6

[17] Bigliardi PL, Alsagoff SAL, El-Kafrawi HY, Pyon JK, Wa CTC, Villa MA (2017) Povidone iodine in wound healing: a review of current concepts and practices. Int J Surg 44:260–268. 10.1016/j.ijsu.2017.06.073 28648795 10.1016/j.ijsu.2017.06.073

[18] Clark WB, Magnusson I, Walker CB, Marks RG (1989) Efficacy of perimed antibacterial system on established gingivitis. (I). Clinical results. J Clin Periodontol 16(10):630–635. 10.1111/j.1600-051x.1989.tb01031.x 2693495 10.1111/j.1600-051x.1989.tb01031.x

[19] Rosling B, Hellström MK, Ramberg P, Socransky SS, Lindhe J (2001) The use of PVP-iodine as an adjunct to non-surgical treatment of chronic periodontitis. J Clin Periodontol 28(11):1023–1031. 10.1034/j.1600-051x.2001.281106.x 11686823 10.1034/j.1600-051x.2001.281106.x

[20] Greenstein G (1999) Povidone-iodine’s effects and role in the management of periodontal diseases: a review. J Periodontol 70(11):1397–1405. 10.1902/jop.1999.70.11.1397 10588505 10.1902/jop.1999.70.11.1397

[21] Hoang T, Jorgensen MG, Keim RG, Pattison AM, Slots J (2003) Povidone-iodine as a periodontal pocket disinfectant. J Periodontal Res 38(3):311–317. 10.1034/j.1600-0765.2003.02016.x 12753370 10.1034/j.1600-0765.2003.02016.x

[22] Xanthopoulou V, Räisänen I, Sorsa T, Sakellari D (2023) Active MMP-8 as a biomarker of peri-implant health or disease. Eur J Dent 17(3):924–928. 10.1055/s-0042-1753454 36063841 10.1055/s-0042-1753454 PMC10569878

[23] Wachtel H, Schenk G, Bohm S, Weng D, Zuhr O, Hurzeler MB (2003) Microsurgical access flap and enamel matrix derivative for the treatment of periodontal intrabony defects: a controlled clinical study. J Clin Periodontol 30(6):496–504. 10.1034/j.1600-051x.2003.00013.x 12795787 10.1034/j.1600-051x.2003.00013.x

[24] Eick S, Straube A, Guentsch A, Pfister W, Jentsch H (2011) Comparison of real-time polymerase chain reaction and DNA-strip technology in microbiological evaluation of periodontitis treatment. Diagn Microbiol Infect Dis 69(1):12–20. 10.1016/j.diagmicrobio.2010.08.017 21146709 10.1016/j.diagmicrobio.2010.08.017

[25] O’Leary TJ, Drake RB, Naylor JE (1972) The plaque control record. J Periodontol 43(1):38. 10.1902/jop.1972.43.1.38 4500182 10.1902/jop.1972.43.1.38

[26] Wickham H. ggplot2: Elegant Graphics for Data Analysis (Use R!). 2nd edition. 2 ed. New York: Springer; 2016.

[27] Marini L, Sahrmann P, Rojas MA, Cavalcanti C, Pompa G, Papi P et al (2019) Early wound healing score (EHS): an intra- and inter-examiner reliability study. Dent J Basel. 10.3390/dj7030086 31480586 10.3390/dj7030086 PMC6784738

[28] Susin C, Fiorini T, Lee J, De Stefano JA, Dickinson DP, Wikesjö UM (2015) Wound healing following surgical and regenerative periodontal therapy. Periodontol 2000 68(1):83–98. 10.1111/prd.12057 25867981 10.1111/prd.12057

[29] Kraft-Neumarker M, Lorenz K, Koch R, Hoffmann T, Mantyla P, Sorsa T et al (2012) Full-mouth profile of active MMP-8 in periodontitis patients. J Periodontal Res 47(1):121–128. 10.1111/j.1600-0765.2011.01416.x 21958332 10.1111/j.1600-0765.2011.01416.x

[30] Schulz S, Zissler N, Altermann W, Klapproth J, Zimmermann U, Glaser C et al (2008) Impact of genetic variants of CD14 and TLR4 on subgingival periodontopathogens. Int J Immunogenet 35(6):457–464. 10.1111/j.1744-313X.2008.00811.x 19046305 10.1111/j.1744-313X.2008.00811.x

[31] Guarnieri R, Reda R, Di Nardo D, Miccoli G, Pagnoni F, Zanza A et al (2024) Expression of IL-1β, IL-6, TNF-α, and a-MMP-8 in sites with healthy conditions and with periodontal and peri-implant diseases: a case-control study. J Dent Res Dent Clin Dent Prospects 18(2):135–142. 10.34172/joddd.40958 39071212 10.34172/joddd.40958 PMC11282203

[32] Flemingson Emmadi P, Ambalavanan N, Ramakrishnan T, Vijayalakshmi R (2008) Effect of three commercial mouth rinses on cultured human gingival fibroblast: an *in vitro* study. Indian J Dent Res 19(1):29–35. 10.4103/0970-9290.38929 18245921 10.4103/0970-9290.38929

[33] Cabral CT, Fernandes MH (2007) In vitro comparison of chlorhexidine and povidone-iodine on the long-term proliferation and functional activity of human alveolar bone cells. Clin Oral Investig 11(2):155–164. 10.1007/s00784-006-0094-8 17216529 10.1007/s00784-006-0094-8

[34] Hamzani Y, Chaushu G (2018) Evaluation of early wound healing scales/indexes in oral surgery: a literature review. Clin Implant Dent Relat Res 20(6):1030–1035. 10.1111/cid.12680 30324746 10.1111/cid.12680

[35] Rojas MA, Marini L, Pilloni A, Sahrmann P (2019) Early wound healing outcomes after regenerative periodontal surgery with enamel matrix derivatives or guided tissue regeneration: a systematic review. BMC Oral Health 19(1):76. 10.1186/s12903-019-0766-9 31064353 10.1186/s12903-019-0766-9 PMC6505273

[36] Mounssif I, Bentivogli V, Rendón A, Gissi DB, Maiani F, Mazzotti C et al (2023) Patient-reported outcome measures after periodontal surgery. Clin Oral Investig 27(12):7715–7724. 10.1007/s00784-023-05362-y 37940683 10.1007/s00784-023-05362-y PMC10713745

[37] Katz J, Melzack R (1999) Measurement of pain. Surg Clin North Am 79(2):231–252. 10.1016/s0039-6109(05)70381-9 10352653 10.1016/s0039-6109(05)70381-9

